# Appropriate fluid resuscitation of septic shock patients pretreated with high doses of catecholamines

**DOI:** 10.1186/2197-425X-3-S1-A225

**Published:** 2015-10-01

**Authors:** JC Lewejohann, M Hansen, H Braasch, C Zimmermann, E Muhl, T Keck

**Affiliations:** University Medical Center Schleswig-Holstein - Campus Lübeck, Surgery - Intensive Care Unit, Lübeck, Germany

## Intr

Appropriate fluid resuscitation for hemodynamic support in septic shock remains a fundamental challenge even for experienced intensivists and should be ideally achieved before administration of vasopressors and inotropes. The development of hemodynamic monitoring revealed that some patients treated with high doses of catecholamines have been improperly resuscitated with fluids before.

## Objectives

The aim of our study is to show the time course of an individualized fluid challenge in septic shock adapted to the patients’ needs resulting in lower catecholamine doses.

## Methods

We analyzed n = 29 pts.; 17 m, 12f; mean age 71 ± 10y [SE]; mean SAPSII 59 [min. 40, max. 87]) with septic shock, mottled-marbled cold extremities and MAP≥65 mmHg on high catecholamines (norepinephrine 16,67µg/min (28 pts.); dobutamine 333,33µg/min (20 pts.); epinephrine 16,67µg/min (17 pts.) [median, range up to 56,67/1666,67/33,3µg/min]) retrospectively after major surgery (university hospital). Median CVP was 17 [5-34] mmHg. Care standard comprised of an extended forced volume challenge combined with active reduction of catecholamines to achieve an adequate fluid loading status, guided by passive leg rising test, clinical signs and in 19 cases by hemodynamic monitoring (PAC, VigilanceII n = 10; FloTrac, Vigileo n = 9, PreSep n = 5 [Edwards-Lifesciences]). It was stopped after clinical improvement with rewarmed extremities, increasing diuresis and lack of improvement by PLR.

## Results

Extended individualized volume challenge consisted of 4.500 ml Ringer [5.250] and 1.000 ml colloids [1.000]. Fluid balance in meantime: +6.465 ml [5.228]. Fluid balances at day

1: +8.820 [5.665];

2: +3.025 [2.785];

3: +1.165 [3.873];

4: +256 [3.266];

5: -388 [2.880];

6: -233 [1.711];

7: + 286 [1.953] ml [median, IQR].

Catecholamine doses were significantly reduced in all pts.: norepinephrine to 0; dobutamine to 166,67; epinephrine to 3,33µg/min (up to 10/666,67/6,67; p < 0,05 [Wilcoxon signed rank test]). Weaning time from catecholamines took 12 h (13,5), afterwards all patients showed rewarmed extremities and a decrease of lactate levels from 2,78 (3,01) to 2,05 (1,68) mmol/l. Hemodynamic constellations were inhomogeneous (CO decreased from 6,3 [1,9] to 6,15 l/min [2,3]; SVR decreased from 995 [220] to 788 [255] dyn*s*cm^-5^) without any cardiac deterioration nor of Pa02/Fi02 (change from 264 [125] to 250 [104] mmHg; median, IQR). Calculation of predicted mortality rate with SAPS II resulted in 66% (min. 25%, max. 96%), 20 patients survived, 9 died.

## Conclusions

Appropriate fluid loading preceding to the use of high catecholamine doses should be a main subject of discussion in patients with severe septic shock, because it is possible to wean quite a few septic shock patients from high dose catecholamines.
